# Audience synchronies in live concerts illustrate the embodiment of music experience

**DOI:** 10.1038/s41598-023-41960-2

**Published:** 2023-10-05

**Authors:** Wolfgang Tschacher, Steven Greenwood, Sekhar Ramakrishnan, Martin Tröndle, Melanie Wald-Fuhrmann, Christoph Seibert, Christian Weining, Deborah Meier

**Affiliations:** 1 University Hospital of Psychiatry and Psychotherapy, University of Bern, Bern, Switzerland; 2 Zeppelin University, Friedrichshafen, Germany; 3 Illposed, Zurich, Switzerland; 4 Max-Planck-Institute for Empirical Aesthetics, Frankfurt am Main, Germany; 5 Applied University for Music, Karlsruhe, Germany

**Keywords:** Autonomic nervous system, Applied mathematics, Human behaviour

## Abstract

A study of 132 audience members of three classical public concerts (all three staged the same chamber music pieces by Ludwig van Beethoven, Brett Dean, and Johannes Brahms) had the goal of analyzing the physiological and motor responses of audiences. It was assumed that the music would induce synchronous physiology and movement in listeners (induction synchrony). In addition to hypothesizing that such synchronies would be present, we expected that they were linked to participants’ aesthetic experiences, their affect and personality traits, which were assessed by questionnaires before and after the concerts. Clear evidence was found of physiological synchrony (heart rate, respiration rate, skin conductance response) as well as movement synchrony of the audiences, whereas breathing behavior was not synchronized. Thus the audiences of the three concerts resonated with the music, their music perception was embodied. There were links between the bodily synchrony and aesthetic experiences: synchrony, especially heart-rate synchrony, was higher when listeners felt moved emotionally and inspired by a piece, and were immersed in the music. Personality traits were also associated with the individual contributions to induction synchrony.

## Introduction

The embodiment of the mind plays an increasing role in cognitive science. In this perspective, cognition is conceptualized as embodied, embedded, extended, and enacted (4E cognition)^1,2^. In the present application, we focused on ‘embodied’, which denotes that the mind is associated with body movement and physiology, on ‘embedded’ (the mind is nested in the affordances of the environment), and ‘enactive’ (the mind is involved in a continuous loop-like exchange between mental prediction and bodily sensory evidence). The embodiment perspective deviates clearly from the traditional ‘cognitivistic’ understanding of cognition as information processing alone or predominantly. This approach has obvious consequences for the field of empirical aesthetics^3^. A large body of (mostly laboratory-based) research has shown that music regularly induces physiological responses in listeners^4–6^. The strong coupling between emotional responses to music and physiological processes is also present in aesthetic experiences of ‘being moved’, which are often linked with physiological phenomena such as chills or tears^7–11^. We therefore focused in the current study on measures of the autonomous nervous system (ANS) and on overt body movement.

The ANS consists of two antagonistic branches, the sympathetic and the parasympathetic branch, which relate to arousal and relaxation, respectively. ANS activation is an example of the circular relationship between mind and body addressed by the embodiment perspective: mental arousal or relaxation has the consequence of bodily arousal or relaxation, whereas the reverse is also true, as the mind becomes alerted by sympathetic physiological activation or relaxed by parasympathetic activation. In analogy to the reciprocal relationship of physiological activation and mental activation, there is also a reciprocity at the level of body movement and so-called body language. An example is gait, where increased vertical movement while walking is found predictive of increased positive mood, and also distinguishes the gait of depressed patients from that of control subjects^12^. In another study on gait, the reverse sequence was explored experimentally: Michalak and colleagues^13^ provided (non-depressed) participants biofeedback that modified their gait patterns on a treadmill, finding that depressed gait favored depressed cognitive styles. Such findings point to a reciprocity between cognition and movement qualities.

A further field of embodiment research concerns the role of embodiment in social interaction. Here, the phenomenon of interpersonal nonverbal synchrony has become a major topic of research: during social interaction people tend to become synchronized and coordinated with respect to their body movement^14^, to their electrodermal activity^15^ and cardiac activation^16^. The synchronization of musicians performing can be viewed as a form of creativity and group flow^17^. Systematic reviews^18–20^ showed that interpersonal synchrony of movement and physiological synchrony were associated with improvements after psychotherapy, as well as with empathy and relationship quality of interactants, suggesting reciprocal links between mind and body also in the context of interpersonal communication. It is established practice in studying nonverbal synchrony to cover the time scale of a few seconds, and thus address the synchrony established in the experienced present moment, the psychological 'now'. It is important to note that in this literature, synchrony is defined statistically, not as perfect alignment or entrainment (see “Method” section for details).

People are commonly unaware of their becoming synchronized. While some findings suggested that highest levels of synchrony may be signs of competition^14^ and even adversity^21,22^, it is predominantly found that synchrony is the signature of prosocial dyadic interactions^23^. We may call this type of synchronization ‘interaction synchrony’ because it emerges directly out of the social interaction itself, in the absence of external stimuli that function as a *zeitgeber*. Interpersonal synchrony in psychotherapy sessions or everyday personal communications, where synchrony arises spontaneously, is pure interaction synchrony. In addition to synchrony as a result of naturalistic social exchanges, some studies have experimentally induced or instructed behavioral synchrony, again finding prosocial feelings and empathy as results of instructed synchrony^24–27^. We may call this type of synchrony ‘induction synchrony’^28^. Induction synchrony is given especially in the setting of classical concerts, where there is little interaction between members of the audience as soon as the performances start. Listeners are not supposed to talk and interact, the audience sits in subdued lighting, and there is little opportunity and motivation for visual contacts in the audience. Thus, if there is evidence of audience synchrony arising in such concerts, it is very likely induced by the music performance and hence induction synchrony. As for the time scale regarded in induction synchrony, the same time scale of a few seconds is meaningful because in that interval aesthetic experiences such as ‘being moved’ occur.

In synchrony studies of listeners' and musicians' body movement measured throughout a live concert^29,30^, significant interaction synchrony was found between musicians^31^, between conductors and musicians^32^, and induction synchrony between the audience members. Physiological synchronies of heart rate, respiration rate and skin conductance response were observed in a different series of classical concerts, and the degree of physiological synchrony was linked with aesthetic appreciation^28^ and musical features^33^. Other studies demonstrated respiratory synchrony in music listeners by studying breathing behavior in detail, beyond just respiration rate, finding respiratory phase alignment in audience members listening to live performed pieces from different genres (renaissance madrigal, romantic, electroacoustic, Japanese popular music)^34,35^. Overall, there is some evidence that music can affect motor and physiological responses in listeners, yet there are still very few synchrony studies, especially in naturalistic settings such as concerts.

The present study was conducted in the context of three live concerts featuring classical music performed by a string quintet. Concerts were organized by the project “ECR—Experimental Concert Research”. We assessed aesthetic experiences of all listeners pertaining to the concerts as a whole and to each of the pieces of the concerts. Additionally, listeners' personality traits were measured prior to the concerts and their affective states before and after the concerts. During the music, physiological responses of audience members were recorded and motion-capture of all listener movements was performed based on video recordings by ceiling cameras in the concert hall. We assumed that listeners' variables would be significantly coupled; thus our first hypothesis was that induction synchrony would be present at the different levels of physiology. Additionally, we expected that the body movement of audience members would be synchronized. Further hypotheses were derived from findings^28^ that such synchronies may be considered proxies of aesthetic immersion in the presented music. In this previous study, the same music was offered, but the audience's physiology was measured per bar, and the concert venue and self-report questionnaires were different. For the present study, we applied comprehensive validated questionnaires, and hypothesized that physiological synchrony would scale with specific personality traits, with listeners’ affectivity and aesthetic experiences, and that this would likewise be true for movement synchrony.

### Hypothesis 1

There is significant audience synchrony of heart rate, electrodermal activity, respiration rate, respiration behavior.

### Hypothesis 2

There is significant audience synchrony of body movement.

### Hypothesis 3

Physiological synchrony is significantly associated with listeners’ personality traits, affectivity and aesthetic experiences.

### Hypothesis 4

Movement synchrony is significantly associated with listeners’ personality traits, affectivity and aesthetic experiences.

## Results

### Descriptive statistics

**Figure 1:**
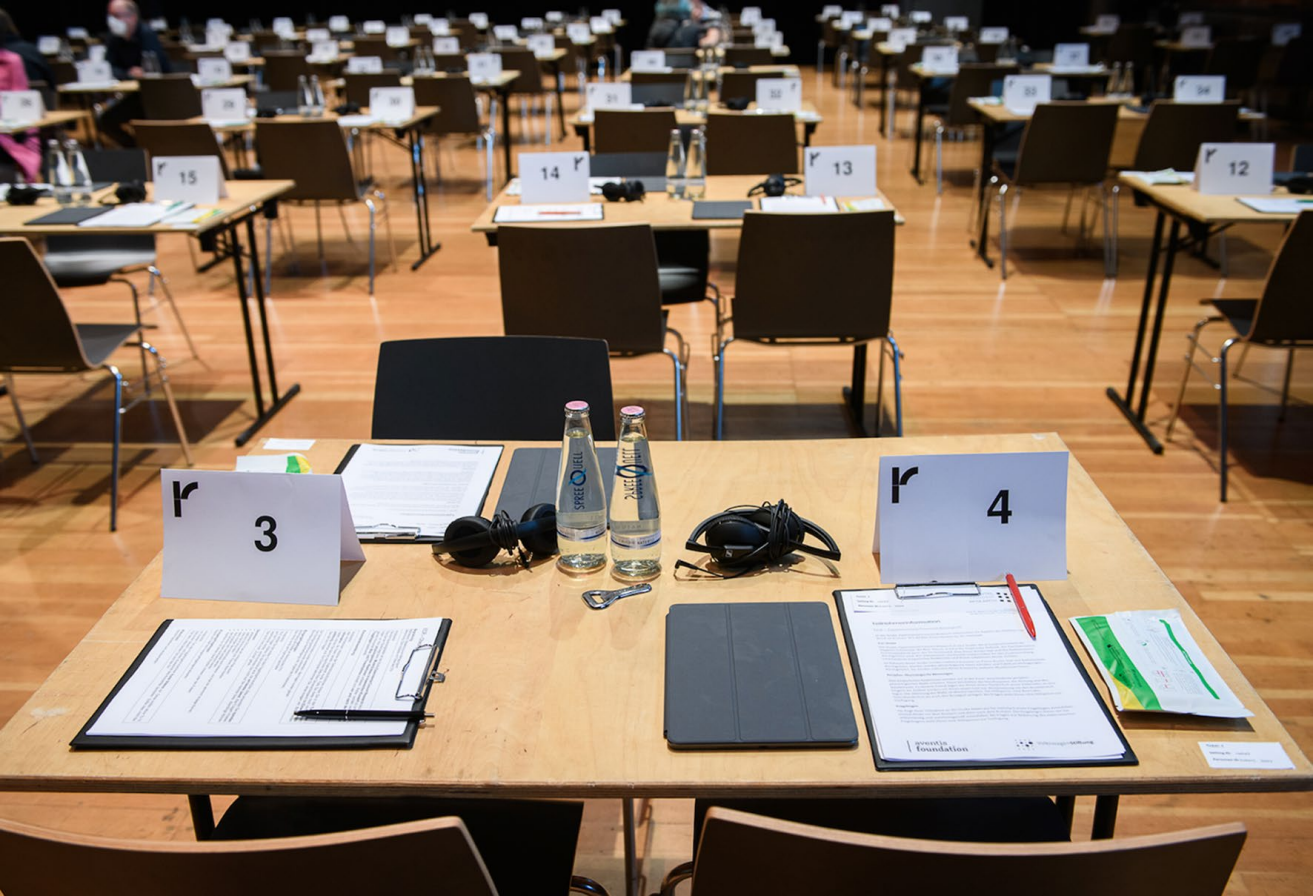
Questionnaires: survey room, numbered tables, written consent forms, questionnaires on iPads. Photo: Phil Dera.

A sample of 132 adult participants with written informed consent visited the concerts. Motion capture was possible in 130 participants, and physiological data were recorded from 123 participants. 125 participants completed both entry and exit questionnaires (Fig. 1). Participants' average age was 46.2 years (range 18–85 years, SD = 17.4). 58.5% of participants were female, 38.5% were male, and 3% preferred not to provide this information or identified as non-binary. Participants reported their highest levels of education as follows: primary/elementary school 3.6%, apprenticeship 1.5%, secondary/high school 11%, university degree in natural sciences/engineering 18.3%, university degree in the humanities/social sciences 41.2%, and university degree in music/arts/cultural studies 24.3%. 94% of participants lived in Germany (in Berlin: 77%). Participants stated their current job to be self-employed/freelancer (16.3%), medium to higher employee (23%), worker or basic employee (12.6%), freelance musician or artist (3%), unemployed (3.7%), in training (1.5%), university student (16.3%), teacher (5.2%), retired (17%), or other (1.4%). Most frequent native languages were German (88.1%) and Spanish (4.4%); the English versions of the entry and exit assessments were used by 12% of participants. Table 1 presents descriptive statistics of the audiences of the three concerts. None of the mean values differed significantly between concerts.

**Table 1:** Descriptive statistics of sample: means (standard deviations) of emotional states, personality traits, movement intensity.

	Concert 1		Concert 2		Concert 3	
	Pre	Post	Pre	Post	Pre	Post
PANAVA-KS	*N* = 46	*N* = 44	*N* = 40	*N* = 41	*N* = 47	*N* = 44
Positive activation (PA)	4.78 (0.88)	4.73 (1.10)	4.84 (1.00)	4.47 (1.13)	4.64 (0.87)	4.61 (0.97)
Negative activation (NA)	2.66 (0.76)	2.37 (0.82)	2.79 (1.19)	2.74 (1.21)	2.87 (1.10)	2.61 (0.87)
Valence (VA)	5.34 (1.24)	5.76 (0.99)	5.48 (1.06)	5.32 (1.17)	5.18 (1.01)	5.57 (0.96)
BFI-10	*N* = 46		*N* = 40		*N* = 46	
Neuroticism	2.70 (0.76)		2.90 (0.88)		3.03 (0.96)	
Extraversion	3.42 (0.86)		3.69 (0.81)		3.29 (0.95)	
Openness to experience	4.10 (0.70)		4.35 (0.62)		4.04 (0.77)	
Agreeableness	3.35 (0.74)		3.48 (0.81)		3.20 (0.72)	
Conscientiousness	3.80 (0.65)		3.76 (0.78)		3.77 (0.70)	
Movement of listeners	*N* = 45		*N* = 41		*N* = 44	
Beethoven	3.01 (23.44)		2.74 (24.98)		3.71 (27.13)	
Dean	3.63 (25.84)		4.74 (32.42)		4.66 (31.86)	
Brahms	5.27 (32.37)		6.24 (36.89)		6.09 (37.95)	

*PANAVA-KS* positive activation negative activation valence short version, *BFI-10* big five inventory-10.

### Physiological synchrony in the audiences

**Figure 2:**
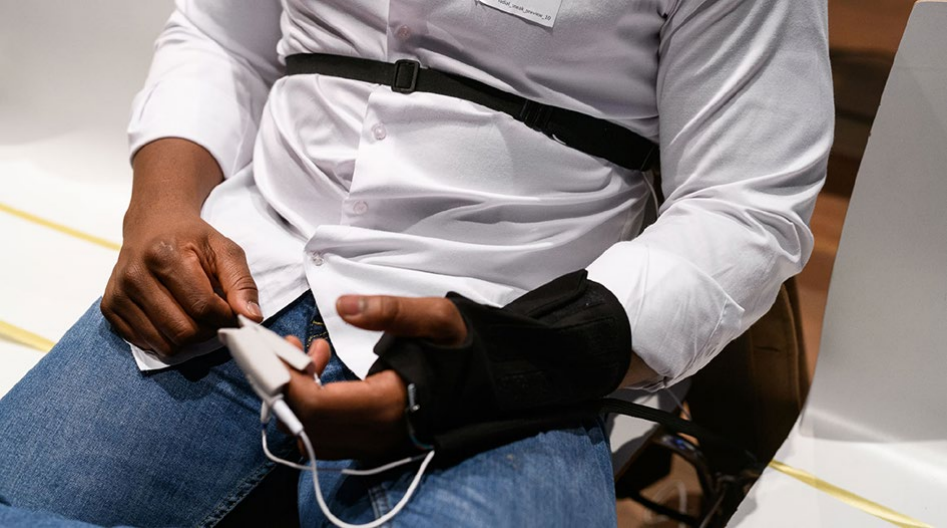
Visitor with data glove, sensors, respiration belt. Photo: Phil Dera.

Physiology of *N* = 123 participants was recorded during the concerts (Fig. 2). The datasets were preprocessed providing these physiological time series for each participant: Heart rate, electrodermal activity, respiration rate and respiration. The physiological data had various degrees of missings for technical reasons: respiration rate, 8.9%; respiration, 14.6%; electrodermal activity, 35%; heart rate, 53.7%. The measures heart rate, electrodermal activity and respiration rate were down-sampled to 1 Hz time series, respiration to 10 Hz. Using the surrogate synchrony (SUSY) algorithm, all dyadic synchronies of the single measures were computed based on these time series and then aggregated per piece and concert. Effect sizes of synchrony against surrogate synchrony were used; such effect sizes are negative in cases of anti-phasic coordination, and positive in in-phase coordination. We analyzed significances of effect sizes (*t*-tests against zero) for each concert, and then each piece, separately. Analyses showed that effect sizes of physiological synchrony against surrogate synchrony were significant in all concerts, except for respiration. Piece-wise synchronies were again insignificant for respiration throughout, yet significant for the other physiological measures with few exceptions (Dean: heart rate concert 1; electrodermal activity in concerts 2 and 3; Beethoven: heart rate concert 3). All synchrony effect sizes of heart rate, electrodermal activity, respiration rate were positive, suggesting in-phase synchrony. Across all concerts and pieces, synchrony was lowest in respiration (M = − 0.02; SD = 0.15); followed by synchrony of electrodermal activity (M = 0.64; SD = 0.87), of heart rate (M = 3.01; SD = 3.98) and of respiration rate (M = 6.85; SD = 9.20). Across all concerts and physiological measures, synchrony was lowest in the Beethoven piece (M = 1.33; SD = 3.08), followed by synchrony in Dean (M = 2.67; SD = 5.69), whereas synchrony was highest in Brahms (M = 4.32; SD = 8.23). In the majority of tests of heart rate, electrodermal activity and respiration rate, hypothesis 1 was supported. This hypothesis was rejected concerning respiratory behavior, which represents not only the rate of respiration but also the detailed timing of inhaling and exhaling. Respiratory behavior was never found synchronized in the audiences. Table 2 provides an overview of physiological synchronies per concert, piece, and measure.

**Table 2:** Physiological audience synchronies per concert and per piece and concert.

	Per concert	Per piece and concert		
		Beethoven	Dean	Brahms
Concert 1				
ES HR (*N* = 18)	3.36	3.95	1.97	4.18
t-test against ES = 0	*t* = 5.12****	*t* = 5.15****	*t* = 1.56	*t* = 3.19**
ES EDA (*N* = 28)	1.02	0.90	1.25	0.91
t-test against ES = 0	*t* = 10.98****	*t* = 4.26****	*t* = 7.96****	*t* = 9.91****
ES RR (*N* = 38)	6.01	2.52	4.34	11.17
t-test against ES = 0	*t* = 9.66****	*t* = 3.41***	*t* = 4.46****	*t* = 8.04****
ES RESP (*N* = 32)	− 0.03	− 0.01	− 0.05	− 0.03
t-test against ES = 0	*t* = − 1.22	*t* = − 0.40	*t* = − 0.94	*t* = − 0.68
Concert 2				
ES HR (*N* = 11)	3.90	3.04	3.70	4.94
t-test against ES = 0	*t* = 4.17****	*t* = 2.34*	*t* = 3.16**	*t* = 2.24*
ES EDA (*N* = 29)	0.27	0.22	0.11	0.47
t-test against ES = 0	*t* = 3.88****	*t* = 3.45***	*t* = 0.60	*t* = 9.04****
ES RR (*N* = 33)	9.41	4.43	9.21	14.60
t-test against ES = 0	*t* = 12.74****	*t* = 6.30****	*t* = 6.72****	*t* = 9.32****
ES RESP (*N* = 35)	− 0.04	− 0.04	− 0.03	− 0.04
t-test against ES = 0	*t* = − 1.56	*t* = − 1.35	*t* = − 0.87	*t* = − 0.74
Concert 3				
ES HR (*N* = 27)	2.40	0.07	4.00	3.15
t-test against ES = 0	*t* = 5.23****	*t* = 0.17	*t* = 4.87****	*t* = 3.08**
ES EDA (*N* = 23)	0.63	0.67	0.41	0.82
t-test against ES = 0	*t* = 5.65****	*t* = 3.59***	*t* = 1.85	*t* = 4.79****
ES RR (*N* = 40)	5.54	1.84	6.10	8.67
t-test against ES = 0	*t* = 9.80****	*t* = 3.05**	*t* = 6.03****	*t* = 7.18****
ES RESP (*N* = 38)	− 0.01	0.01	− 0.02	− 0.01
t-test against ES = 0	*t* = − 0.31	*t* = 0.67	*t* = − 0.69	*t* = − 0.16

Varying sample sizes *N* due to missings.

*ES* effect size, *HR* heart rate, *EDA* electrodermal activity, *RR* respiration rate, *RESP* respiration.

*p < 0.05; **p < 0.01; ***p < 0.001; ****p < 0.0001.

### Movement synchrony of the audience

On the basis of motion capture of individual whole-body movement, movement synchrony was computed across *N* = 130 concert listeners for each concert and piece separately, using multivariate surrogate synchrony (mv-SUSY^36^). For concert listeners, concert-wise analyses showed that empirical synchrony of movement significantly exceeded surrogates in all concerts. Piece-wise synchrony analyses generally supported these results, indicating above-random synchrony effect sizes across pieces, with the exception of Beethoven in concert 1. In this case, empirical synchrony was higher than surrogate synchrony, yet this difference did not reach significance. In Table 3, findings for concert listeners are presented. In sum, the majority of results supported hypothesis 2 on the presence of above-random movement synchrony of the concert audiences.

**Table 3:** Movement synchronies of concert listeners per concert and per piece.

	Per concert	Per piece and concert		
		Beethoven	Dean	Brahms
Concert 1				
Empirical synchrony λmax	8.99	8.16	8.61	9.56
Surrogate synchrony λmax-surr	8.62	7.98	8.22	9.14
ES (mv-SUSY)	0.29	0.13	0.33	0.32
Test λmax > λmax-surr	*t* = 4.60****	*t* = 1.51	*t* = 3.78***	*t* = 3.60***
Concert 2				
Empirical synchrony λmax	9.74	9.12	9.99	9.84
Surrogate synchrony λmax-surr	8.76	8.71	8.78	9.31
ES (mv-SUSY)	0.73	0.39	0.99	0.36
Test λmax > λmax-surr	*t* = 12.49****	*t* = 3.34**	*t* = 10.38****	*t* = 4.94****
Concert 3				
Empirical synchrony λmax	9.19	8.70	8.70	9.45
Surrogate synchrony λmax-surr	8.61	8.28	8.25	9.12
ES (mv-SUSY)	0.44	0.35	0.38	0.24
Test λmax > λmax-surr	*t* = 4.92****	*t* = 2.89**	*t* = 4.37****	*t* = 2.88**

*p < 0.05; **p < 0.01; ***p < 0.001; ****p < 0.0001.

### Synchrony and personality traits

Synchrony of whatever type is a measure of interpersonal coordination, signifying two or more individuals. Thus synchronies denote a data level different from measures of self-report, which were obtained individually by rating scales. For this reason, we developed a method by which synchrony can be directly linked with self-report items: the concept of synchrony contribution^37^. Synchrony contribution values denote how much each individual listener contributed to the collective synchrony of the respective audience. Synchrony contributions were available for each physiological variable and for body movement. Respiration behavior was not considered further because it was not found synchronized. The synchrony contribution values served as the dependent variables in step-wise regression models (backward regression) that included the data of all three concerts. Predictors were listeners' personality factors of the 'Big-Five' scales. The resulting models depicted in Table 4 show those models that obtained the best fit in terms of Akaike's information criterion (AIC). It was found that 'Openness to new experiences' and 'Agreeableness' were significantly associated with synchrony, whereas 'Neuroticism' and ‘Extraversion’ were associated negatively. These findings are in support of hypothesis 3. Electrodermal activity yielded no AIC-optimal model, and the null model is shown in Table 4. The model for movement synchrony also tended towards an association of synchrony with 'Openness', but this did not reach significance (thus no support of hypothesis 4).

**Table 4:** Synchrony contribution and personality traits.

Predictors	Synchrony contribution of …							
	RR		HR		EDA		Movement	
	Estimate	*t*	Estimate	*t*	Estimate	*t*	Estimate	t
Intercept	5.57	1.45	− 2.54	− 0.88	0.64	9.07****	− 0.03	− 1.83
Extraversion			− 0.93	− 2.52*				
Agreeableness			1.55	2.73**				
Conscientiousness								
Neuroticism	− 2.00	− 2.99**						
Openness	1.73	2.16*	0.88	1.71			0.01	1.87
*N*	109		55		80		129	
Rsquare	0.11		0.21		0.00		0.03	
Whole model test	*F* = 6.65**		*F* = 4.58**		–		*F* = 3.48	

Results for stepwise backward regression of synchrony contribution predicted by personality traits.

*HR* heart rate, *EDA* electrodermal activity, *RR* respiration rate, *N* available data across all three concerts, *Rsquare* explained variance.

*p < 0.05; **p < 0.01; ****p < 0.0001.

### Synchrony and aesthetic experience of pieces

Nine AESTHEMOS items were available in the exit questionnaire for the evaluation of each of the three pieces of the concerts. The mean ratings of pieces were significantly different on most of these scales: the contemporary Dean piece was assessed significantly less likable, less known, more annoying, less moving, less melancholic, and less happy. With respect to 'boring' and 'inspiring', the three pieces were experienced not significantly different. To reduce the intercorrelations of items for subsequent regression analysis, items were factorized in three factors. 'F1-inspiring' represented items such as "inspired me", "was interesting" and "moved me emotionally". 'F2-well-known' stands for "was known to me" and "made me happy", and 'F3-annoying' for items "annoyed me" and "made me melancholic".

Stepwise backward regression models were computed, in each piece, for physiological synchronies (heart rate, electrodermal activity, respiration rate; respiration behavior was excluded) and for movement synchrony. Insignificant predictors of the resulting step-wise models were discarded until only models with significant predictors remained. These are depicted in Table 5. Two significant models were found for the Dean and Brahms pieces; in both, heart rate synchrony was positively linked to the factor 'F1-inspiring'. The piece by Beethoven was not significantly associated with assessments. Hypothesis 3 was partially supported for aesthetic assessments at the level of pieces, hypothesis 4 was not supported.

**Table 5:** Synchrony and piece assessments. Results for stepwise backward regression of synchrony contribution (dependent variable) by aesthetic piece assessments and piece.

Predictors	Synchrony contribution heart rate	
	Estimate	*t*
Dean		
Intercept	3.49	5.42****
F1-inspiring	1.37	2.40*
F2-well-known	*–*	*–*
F3-annoying	*–*	*–*
*N*	48	
Rsquare	0.11	
Brahms		
Intercept	3.50	6.53****
F1-inspiring	1.42	2.25*
F2-well-known	*–*	*–*
F3-annoying	*–*	*–*
*N*	51	
Rsquare	0.09	

Rsquare, explained variance.

*p < 0.05; ****p < 0.0001.

### Synchrony and aesthetic experience of concerts

Factor analysis summarized concert experience, which had been measured by 18 single items of the exit questionnaire. The factors were labeled 'F1-immersion', 'F2-part of audience', 'F3-in company', 'F4-reflecting', and 'F5-feeling disturbed'. As in the models on piece experiences, stepwise backward regression models were computed for the physiological synchronies (with respiration excluded) and for movement synchrony. The resulting best models according to AIC are depicted in Table 6. Synchrony contributions of heart rate were associated with immersion experiences, and less so in concert 3. Electrodermal synchrony was lower in listeners who experienced a good time with companions ('F3-in company'), and there were also differences between concerts. Synchrony contributions of movement and respiration rate were not significantly linked to aesthetic experiences concerning the whole concerts, and hypothesis 3 was partially supported.

**Table 6:** Synchrony and concert experience. Results for stepwise backward regression of synchrony contribution (dependent variable) by aesthetic concert assessments and by concert.

Predictors	Synchrony contribution of…							
	RR		HR		EDA		Movement	
	Estimate	*t*	Estimate	*t*	Estimate	*t*	Estimate	t
Intercept	7.59	11.76****	2.93	9.20****	0.63	10.76****		
F1-immersion			1.31	3.26**			–	
F2-part of audience							–	
F3-in company					− 0.23	− 3.48***	–	
F4-reflecting							–	
F5-feeling disturbed							–	
Concert 1	− 1.82	− 2.83**			0.33	4.10***		
Concert 2					− 0.33	− 4.17****		
Concert 3	− 1.82	− 2.83**	− 0.76	− 2.35*				
*N*	111		55		77		131	
Rsquare	0.07		0.21		0.36		–	
Whole model test	*F* = 7.99**		*F* = 7.03**		*F* = 13.40****		–	

*HR* heart rate, *EDA* electrodermal activity, *RR* respiration rate. Rsquare, explained variance.

*p < 0.05; **p < 0.01; ***p < 0.001; ****p < 0.0001.

### Synchrony and affectivity

Affectivity was assessed before and after the concerts using the PANAVA-KS, with the scales 'Positive activation', 'Negative activation' and 'Valence'. We computed multiple regression models to predict the four synchrony contribution measures (heart rate, electrodermal activity, respiration rate; movement) by the affect scales before the concert. No significant model or predictor was found. The same was true when affect after the concert was predicted by the synchrony contribution measures. Repeating analyses using stepwise regression models likewise entailed no significant prediction of synchrony by prior affect, nor prediction of post-concert affect by synchrony. Therefore, hypotheses 3 and 4 were rejected for affectivity.

## Discussion

The present sample comprised 132 concertgoers, who visited one of a series of three public concerts that staged classical music for string quintet. The basic goal of this study was to determine whether exposure to the concert music entailed listeners' synchronization of physiological and behavioral dynamics, so-called audience synchrony. The second goal was to explore the expected associations between the individual degree of 'being in sync' and individual psychological variables such as personality traits, affective states, and aesthetic experiences during the concerts.

Concerning the first goal, substantial evidence of physiological and movement synchrony between audience members was found. Thus the audiences of the three concerts resonated with the music, their music perception was embodied. The music had significant effects on listeners' heart rate, respiration rate, electrodermal activity and overall body movement. This resonance however did not go as far as synchronizing listeners' breathing beyond entraining their breathing frequencies; respiration in the sense of inhaling and exhaling behavior was not found synchronized. The significant synchrony of body movement appears noteworthy, as the audiences of all concerts were seated in dimmed lighting; in addition, large distances between seats had to be observed owing to the pandemic Covid restrictions in Germany at the time. The synchronies are therefore presumably 'induction synchronies' because they are unlikely to rest on interactions between the participants. Thus, hypothesis 1 was supported for all physiological measures, but rejected for respiration. Hypothesis 2 was supported.

As synchronies were induced by the music, these synchronies may contain information on the psychological relations between listeners and the music. Exploring the associations between embodied synchrony and psychological variables was the second goal of this study. Associations suggested a pattern of personality traits that contributed to more synchrony (Agreeableness and Openness). Thus, trusting, sociable, imaginative persons who were interested in art were more prone to become synchronized. Other personality styles reduced being in-sync (Neuroticism and Extraversion), which means that more nervous, insecure persons, and also outgoing extraverted people became less synchronized by the music.

No indications were found that the participants' affectivity influenced their synchrony, which is possibly due to the momentary nature of the affective states (in the entry and exit questionnaires, the affectivity items were introduced by "How do you feel right here and now?"). The state-character of affectivity may not have generated much impact on subsequent experiences during music listening, and hence on synchronizing with the music.

The third group of items was specifically directed towards aesthetic experiences to each of the three pieces. A number of associations between experiences and synchronies were found, pointing towards embodied experiencing of music. Individual contributions to audience synchrony were higher when a piece moved listeners emotionally and inspired them. These links were however only found in heart-rate synchrony (Table 5).

Concert assessments of being immersed and emotionally moved were again linked to heart-rate synchrony, which was analogous to the responses to pieces. Electrodermal synchrony was attenuated in people who estimated the social aspects of concerts, such as being in company and meeting friends. This may indicate that the music experience did not have first place in participants rather interested in socializing, leading to a decrease of their contributions to audience synchrony. This finding was also in line with the role of extraversion as a personality style.

Hence, hypothesis 3 of the present study was partially supported, whereas hypothesis 4 concerning movement synchrony was not supported. Findings may be viewed as a successful replication of the few existing previous reports of significant audience synchrony in live classical concerts, that shared with the present study an emphasis on naturalistic conditions instead of laboratory environments^28,29^. Importantly, two methodological innovations were introduced in the present analysis. The first is the methodology of assessing multivariate synchrony, which appears especially appropriate when studying social aggregates beyond the dyad, such as audiences of concerts. The second methodological step forward in our opinion is the concept of synchrony contribution, which sidesteps the problem of connecting synchrony, by definition an interpersonal construct and variable, to psychological and experiential variables that are intrapersonal and assessed individually. Synchrony contribution allows to relate the two types of variables more directly and thereby simplifies analysis because hierarchical multilevel statistics as in^28^ are no longer demanded.

Limitations were encountered with respect to the physiological recordings. There is a trade-off between non-invasiveness of physiological recordings and data quality, and the present study emphasized the wearing comfort and non-intrusiveness of physiological sensors over data quality. This compromised especially the photo-plethysmographic detection of heartbeat, which was prone to failure and caused a high number of missings. Post-hoc analyses showed no significant associations of heart-rate missings with individual contributions to motion synchrony, or to participants' affect, personality and age. There was however a significantly higher proportion of missings in female participants, possibly due to anatomical differences. Consequently, data loss may not have occurred entirely at random, and missings also limited the power of the respective tests. Future concert research should therefore consider reducing the measurement problems by focusing on better placement of sensors that detect blood volume pulse and on solid fixation of electrodes to measure skin conductance.

## Method

### Participants and setting

In the context of the Experimental Concert Research (ECR) project (www.experimental-concert-research.org) three public concerts were organized between September 10 and 12, 2020, at the concert venue Radialsystem in Berlin, Germany. Owing to pandemic Covid regulations, the seats were not fully occupied to keep physical distances between attendees. Concerts were promoted via the regular channels of the Radialsystem as well as on social media. Prior to purchasing tickets, visitors were informed that the concerts were part of the ECR project and invited to participate in the project. A study description was handed out that informed about all forms of data acquisition. 141 concert attendees provided written informed consent to take part in the study. We excluded seven participants with age below 18 and two participants who did not attend the concerts (drop-outs after entrance questionnaire). The current analyses are based on 132 participants. The experimental procedures were previously approved by the Ethics Board of the Max Planck Society. All research was performed in accordance with the Declaration of Helsinki and the relevant guidelines and regulations in Germany.

### Stimuli and procedure

All concerts presented the same program of chamber music for string quintet, with pieces by Ludwig van Beethoven (op. 104 in C minor), Brett Dean (Epitaphs) and Johannes Brahms (op. 111 in G major). The three pieces represented different musical styles (Viennese classical, Contemporary, Romantic, respectively). The Yubal ensemble played the first two concerts. The last concert was performed by Ensemble Epitaph. On average, concerts lasted for approximately 1 h and 15 min. Upon arrival, project assistants welcomed the concert attendees who had signed the informed consent and lead them to desks where they filled out an 'entrance questionnaire' before entering the concert hall (Fig. 1). Questionnaires were available in either German or English. When seated in the concert hall, assistants attached the sensors for physiological recording. During each concert, ten birds-eye cameras covered the stage and the seating area. Cameras were fixed at approximately 5 meters above the ground. The birds-eye perspective allowed to capture individual body movement in the audience. All recordings were controlled and synchronized with the help of a central dashboard. After the concert, participants completed the 'exit questionnaire'.

### Self-report measures

The two questionnaires comprised multiple scales and items for the assessment of subjective concert experience. For the present analysis, we used standardized measures of participants' affectivity, personality traits, and aesthetic experiences, as well as demographic information. Affective states were measured before and after each concert using the 'Positive Activation Negative Activation Valence' scale, short version (PANAVA-KS^38^). The PANAVA-KS comprises ten items forming three subscales: positive activation (PA), negative activation (NA), and valence (VA). PANAVA-KS items are bipolar. For example, the first item comprises the poles ‘tired’ and ‘wide awake’. The PANAVA-KS subscales have demonstrated good internal consistency with Cronbach’s α ranging from 0.74 to 0.83. To assess the 'Big-five' personality traits, we applied the 10-items 'Big Five Inventory' (BFI-10^39^). Participants rated the BFI-10 based on five-point Likert scales. BFI-10 subscales are Extraversion, Agreeableness, Conscientiousness, Neuroticism, and Openness assessed by two items each. BFI-10 scales were shown to represent the Big Five structure or the original Big Five Inventory well, have substantial convergent and discriminant validity^39^).

Aesthetic experiences were assessed by participants in the ‘exit questionnaire’. Overall concert experience was assessed with a battery of 18 five-point scales^40^. We conducted an ad-hoc factor analysis with Quartimin oblique rotation to reduce the dimensionality of the 18-items battery to five factors, with explained 82% of the variance. We labeled the factors 'F1-immersion' (concentrating fully on the music, being emotionally moved), 'F2-part of audience' (feeling connected), 'F3-in company' (having a good time with one’s companion, meeting friends), 'F4-reflecting' (concert made me think), and 'F5-feeling disturbed' (disturbed by noises or else).

Experiences when listening to the single pieces were assessed by nine six-point scales per piece, which were based on items of the Aesthetic Emotions Scale (AESTHEMOS^41^). The items were: "The piece…" "… I liked" (item 1); "… was known to me" (item 2); "… annoyed me" (item 3); "… moved me emotionally" (item 4); "… was interesting" (item 5); "… inspired me" (item 6); "… made me melancholic" (item 7); "… made me happy" (item 8); "… bored me" (item 9). As the items were considerably intercorrelated, we again conducted factor analysis with Quartimin rotation. Factor analysis generated three factors, which explained 65.2% of the variance of items. Factors were labeled F1-inspiring, F2-well-known and F3-annoying. F1-inspiring subsumed items "inspired me", "was interesting" and "moved me emotionally". F2-well-known loaded on "was known to me" and "made me happy". F3-annoying was determined by items "annoyed me" and "made me melancholic".

### Physiological measures

When participants were seated in the concert hall, assistants attached a glove equipped with sensors to collect physiological measures (blood-volume pulse, electrodermal activity) and a respiration belt to measure breathing (Fig. 2; devices were produced by biosignalsplux, PLUX Wireless Biosignals, S.A.). Physiological data were acquired at 200 Hz sampling rate and were processed using the BioSPPy library^42^) to extract higher-level signals from the device-output signals (e.g., heart rate from blood-volume pulse, respiration rate (RR) from belt displacement, or skin-conductance response (SCR) from electrodermal activity).

Electrodermal activity (EDA) was measured from electrodes attached to two fingers of the non-dominant hand, and pre-processed using Ledalab^43^. As we were interested in event-related responses to the music, only phasic SCR was used in the present analysis of EDA, and the signal was down-sampled to 1 Hz time series.

Heart rate (HR) was derived from the blood volume pulse captured by a photo-plethysmographic sensor over one fingertip. The instantaneous HR signal computed by BioSPPy was converted to continuous HR by linear interpolation and saved as 1 Hz time series.

Respiration activity (RESP) was measured by a strain-sensitive belt placed over clothing beneath the thorax. Respiration data were low-pass filtered (0.6 Hz), demeaned and down-sampled to 10 Hz time series. Additionally, RR was derived from the data, again using the BioSPPy library, and, as with HR, the instantaneous RR signal was converted to continuous 1 Hz signal.

### Motion capture

**Figure 3:**
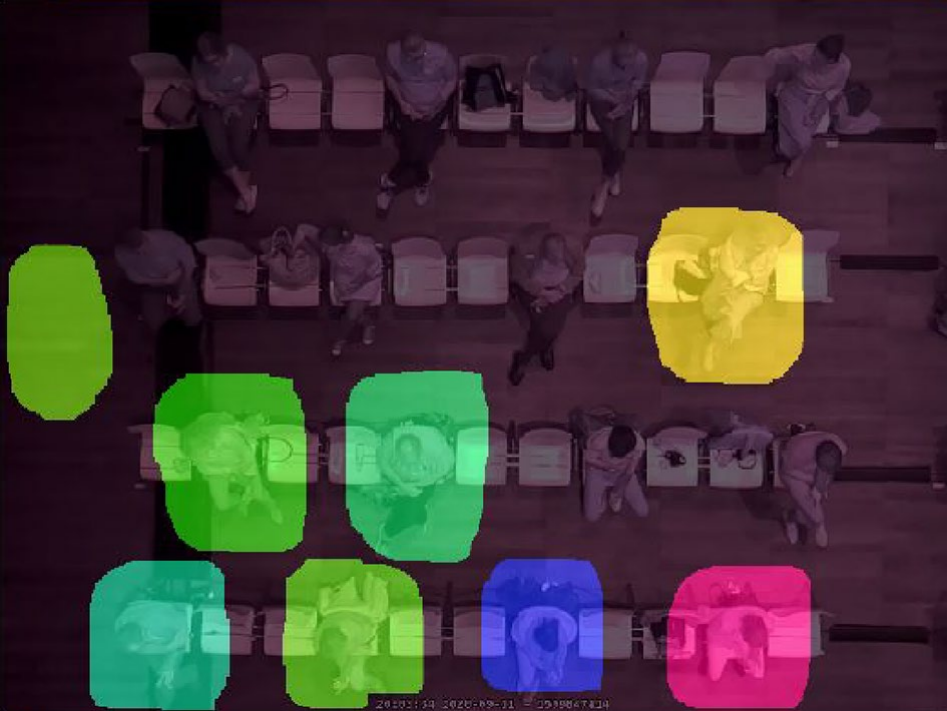
Principle of movement capture from top-view video using Motion Energy Analysis. The shaded areas define the regions of interest within which all pixel changes were detected to operationalize body movement.

Motion Energy Analysis (MEA) is a method to detect movement in video recordings^44^. MEA was inspired by the approach of Grammer and colleagues^45^, who derived the extent of body movement from the number of pixel changes from frame to frame of a digital video. Pixel changes can be quantified within a specific 'region of interest' (ROI) of the video (Fig. 3). Here, ROIs were chosen in the various birds-eye videos that together contained all audience members. The viewing angles from above allowed to define ROIs that covered the pixel changes caused by each individual participant to yield a measure of the overall body movement caused exclusively by each individual, without overlaps. MEA Version 3.06 was applied to the MPEG-4 video recording with 15 frames per second, generating 15 Hz time series.

### Surrogate synchrony (SUSY)

In general, synchrony means that processes are, or become, coordinated and coupled at a level exceeding chance. The data are thus documented as time series. Most methods of synchrony computation function within the time domain, using the cross-correlation function of two time series. Other methods are based on the frequency spectrum of processes or on measures of information^46^. We developed two different correlation-based algorithms, the surrogate synchrony approach (SUSY, cf. www.embodiment.ch; R-package^47^), which is bivariate, and the multivariate surrogate synchrony approach (mv-SUSY). These two algorithms were implemented in the present study.

Different definitions of synchrony are possible. Especially in musical terms, one may consider as 'synchronous' only such phenomena where processes are exactly or almost exactly aligned, without much of a temporal delay ('lag'). Even short lags between signals would thus appear to be 'out of sync'. The statistical synchrony approach of SUSY, which is used in the present study, however tolerates and includes lags because people may have different response times with regard to musical stimuli (positive lags), and may also differ with regard to their anticipation of musical events (negative lags). The present definition of synchrony is statistical because in a second step of SUSY the differently lagged cross-correlations are tested against a control condition, the randomly allocated surrogates. In the psychological field of nonverbal synchrony, terminology is still inconsistent: attunement, concordance, coupling, entrainment, mimicry, coordination, adaptation, synchronicity, and more. 'Synchrony' (Greek: 'together in time') is the increasingly prevailing term to denote the phenomenon as this term makes no causal assumptions, does not claim intentionality, is semantically neutral and not by convention used in other contexts.

In SUSY the cross-correlations are computed segment-wise; time series are cut into segments of e.g. 30 s duration, and the cross-correlations within each segment are computed across a certain range of lags *L*. A default value in many studies is choosing maximum lags up to  ± 3 s or ± 5 s, so that all cross-correlations in a 6- or 10-s window, the cross-correlation range *L*, are considered. Segment-size and window-size are basic parameters in SUSY. This operationalization thus includes the simultaneous (*L* = 0) correlation as well as time-lagged (cross-)correlations. To allow aggregation, all cross-correlations must be transformed using Fisher’s *Z* transformation. A general measure of synchrony is then mean *Z*, aggregated inside each segment and then across all segments of the time series. In the present study we used exclusively the non-absolute values of *Z*, which allows to distinguish between in-phase (*Z* > 0) and anti-phase synchrony (*Z* < 0); anti-phase means that one person's values may be consistently high whenever the other person's are low. In this study we used segment size of 30 s and lags of ± 5 s. Thus, the cross-correlation range was *L* = 10 s, which means that the psychologically significant 'moment' of several seconds, the psychological 'now', is covered. This timespan contains between three to seven musical bars, depending on the piece. Thus, as we may assume that phrase-length of the music is commonly four bars, *L* approximately covers a musical phrase. The choice of segment size takes care of possible non-stationarity of the time series such as slow changes of means or variances. Importantly, obtaining a *Z* value for each segment allows to control for random cross-correlation by surrogate analysis.

The second step in SUSY consists of surrogate tests^48^. Surrogate time series constitute the control condition for mean *Z*. We generated surrogate time series by randomly shuffling the sequence of all segments of a time series. From a dyadic time series with *n* segments, *n*(*n* − 1) different surrogates can be produced, each of which entails pseudo correlations, as their sequence is falsely arranged, whereas the means, distributions, and autocorrelations of the original time series are preserved. If there were significant trends in the measured time series, these are also destroyed by segment shuffling.

The surrogate step finally generates a signature of synchrony, namely the effect size ES, for non-absolute *Z* cross-correlations, defined as the difference of the ‘real’ *Z* and the mean of all surrogate *Z*, divided by the standard deviation of the surrogate *Z*. Thus, ES is an effect size at the level of the single bivariate process. Multi-person settings, for example an audience, may be described by the ensemble of all dyadic synchronies between the individuals of the audience, hence the mean value of all dyadic synchronies within the audience.

mv-SUSY (multivariate Surrogate Synchrony^36^) is an extension of the dyadic synchrony algorithm SUSY^47^, and in the present analysis was applied solely to the movement time series. The algorithm mv-SUSY allows to assess the overall synchrony of multivariate datasets, here of all *m* members of an audience, in one step instead of aggregating all dyadic synchronies. Synchrony is estimated by the amount of coupling between (not within) the multiple time series. mv-SUSY was previously introduced and validated based on simulated and empirical time series datasets^36^. This multivariate extension of correlational approaches utilizes the eigenvalues of the correlation matrix of the m-dimensional time series. Here we applied the λmax (lambdamax) method of mv-SUSY, which is based on the eigendecomposition of the *m* × *m* correlation matrix of the multivariate time series data^49^. The largest eigenvalue of this matrix, its λmax, is the ratio between the largest eigenvalue and the sum of all eigenvalues, which positively scales with the amount of coupling between the single elements of the multivariate dataset.

To control for spurious results of the mv-SUSY procedure, we apply surrogate analysis by segment shuffling as in SUSY. The time series of the complete data, for example that representing all listeners of a concert, is cut into non-overlapping segments (here with segment size 5 s), and λmax is computed in each segment. The value of ‘real’ synchrony is aggregated from the mean of all the segment synchronies. To generate surrogate datasets, the segments of all *m* single time series are randomly permutated ('shuffled'), and the same eigendecomposition procedure is applied on these shuffled surrogate datasets. Permutation can be repeated many times (here, 1000), and the resulting surrogate synchrony is thus the mean of the 1000 surrogate lambdas, termed λmax-surr. Finally, the effect size (ES) of multivariate synchrony is given, as in SUSY, by the difference between empirical synchrony λmax and the mean surrogate synchronies λmax-surr, divided by the standard deviation of the 1000 surrogate synchronies.

### Significance of synchrony

We assessed hypotheses 1 (heart rate, electrodermal activity, respiration rate, respiration behavior is synchronized in audiences) and 2 (body movement is synchronized in audiences). The synchrony values were tested for significance per concert and per piece within each concert. For hypothesis 1, this was accomplished by testing the mean effect sizes ES of all dyadic combinations of participants against the value ES = 0 (0 means there is no synchrony), using one-sample *t*-tests. For hypothesis 2, we tested whether λmax exceeded the surrogate lambdas λmax-surr, again with one-sample* t*-tests.

### Synchrony contribution

The audience synchronies, based on all dyadic synchronies in the case of SUSY, or based on the total of all participants in the case of mv-SUSY, are dyadic or multi-personal in nature. For the sake of linking synchrony to individual self-report variables, we computed individual synchrony contribution values that represent how much each individual participant contributes to the overall audience synchrony in each of the three concerts. In SUSY, synchrony contribution of participant X is computed as the mean ES of all dyadic combinations of X with all other members of the audience. For example, in an audience of 40 listeners, participant X's synchrony with regard to all 39 other participants in this audience is computed, and X's synchrony contribution is thus the mean of these 39 dyadic values. In mv-SUSY, synchrony contribution of participant X is defined by comparing the ES of the complete audience (EStotal) with the ES of the same audience without considering participant X (EStotal–X). Synchrony contribution of participant X is then the difference EStotal–EStotal–X. Measures of symmetry and dispersion of the distributions of individual synchronies may indicate differences between the three audiences. In the mv-SUSY subset, we tested skewness and equality of variances finding similar skewness and standard deviations of synchrony contributions across concerts. Levene’s test for equality of variances was insignificant (*F* = 1.83, *p* = 0.16).

### Associations between synchrony and self-report variables

We used the same regression approach to detect associations between synchrony contribution (of physiological measures and of movement) and self-report variables (personality traits, aesthetic assessments of music pieces, aesthetic assessments of concerts, affect scores). This approach addressed hypotheses 3 (physiological synchrony is linked to self-report) and 4 (movement synchrony is linked to self-report). Hypothesis 3 included only those physiological measures that indicated significant synchrony in the tests of hypothesis 1. The regression method was step-wise backward regression with the rule of stopping at the model that produced the minimum of Akaike's information criterion (AIC), pointing to the best-fitting model. When the stepwise models contained nonsignificant predictors, these were deleted, and the model again computed. All procedures were performed using JMP Pro 15.1 software (SAS Institute Inc.).

## Data Availability

Data are available upon request to the first author.
